# Development of Dental Tissues

**Published:** 1863-07

**Authors:** W. H. Atkinson


DENTAL REGISTER OF THE WEST.

VOL. XVII.] JULY, 1863. [No. 7.

### Original Essays and Communications.

### DEVELOPMENT OF DENTAL TISSUES.

(Read before New York Society of Dental Surgeons.)

BY W. H. ATKINSON.

NUMBER I.

To get at any clear, concise textual understanding of any structure, it is important to call to our aid some mind which has already comprehended the synthesis and analysis of the particular structure proposed to be considered (studied together.)

And as the architect must be in his individual capacity equal to all the materials and workmen who erect the building, *theory* is proved to be at least the first half of every perfect work of creation, whether it be cell or system, cot or mansion, the smallest syphon or the most immense aqueduct!

This potent, unseen agency, represented by the 0, is always the primal step in every creative act and is called type, theory, draft, design and a host of other names all indicating this first step toward the putting on of corporeal or what has been denominated "real" presence.

The how of this first complete step, equivalent to all the subsequent steps to full expression of bodily form of material presence, is too occult for elucidation by the use alone of any language now at command, but when a truly natural language shall find means of expression any feeling, idea, thought, opinion, belief or knowledge, will be easy of complete enunciation.

But because we cannot anticipate the blessedness of the coming times and at once tell all that we can desire to communicate from the treasurer of our mental workshops, it is no reason why we should not essay to take a few primal steps in that direction.

One word of argument in proof of the assertion that the (0) nought equals all things, or that "nothing is equal to something."

I will state that the fetus is composed of seen and unseen elements; now the seen are the materials and workmen who labor together, under the direction of the unseen elements, thus producing the exact symbol of the unseen reality in form, size, shape, &c., &c., precisely in the ratio of agreement between the workmen and the type, perfection of development always being the index of the harmony of these, while imperfection is as surely that of inharmony.

This suggests the query what harmony and its opposite really are and how we may *know* them.

The answer to this query will be also the solution of very many more, and is the exact balance of forces, which is known by the agreeableness of diversity in similarity.

I am aware of the poverty of the language here used, which is another proof that our needs of a perfect language are upon us demanding one from the infinite source of all forms of life and being, which is positive proof that it is fast coming.

Now the ovum is recognized as a fetus so soon as it is enclosed in the amnion, which is long before even the rudiments of any of its teeth are visible under the best glasses; but theoretically, ideally and really they are all there in type; which will be proved in the case of each one that is evolved, under the law of procedure of development, to even the most obtuse vision.

Now teeth, and in fact all bodies, in passing from the typal or theoretic, unseen state of existence demand that the seen elements which are to enter into them must approximate the typal state of unseenness or invisibility, fineness of attenuation, solution or approximation to a spiritual state, so that the difference shall be but in degree of life resident therein, and the pure life which awaits this condition to enable it to take up its residence within this solution of apparent or basal material elements as fast as it becomes organized and solidified in accordance with the form, the unseen type or real tooth, or other body, has put on in its typal or theoretic condition.

This is proven to us as soon as we take time to reflect that all the soluble elements of the teeth circulate in one homogeneous mass of blood, that is poured alike into the sacs of incisor, canine, bicuspid and molar, and if the directing agency had its residence in the blood there could be no difference in shape or other character of the various classes of teeth other than size in accordance with the quantity of materials brought to the sacs.

Therefore we know that the agent set over the building of the various teeth *must* have its residence in the sacs or the molecules composing them, or there could be no stated limit or permanent regularity of form to the teeth, and we would be liable to have a heterogeneous product that never knew when it had grown enough or grown rightly.

All the seen or apparent elements out of which teeth are evolved originate in the blood of the organism in which they occur, and are complicated in accordance with the degree of elevation in the scale of the particular being in possession of them; low and simple creatures having teeth of few elements and highly organized ones more complicated dental structures.

So diversified are the teeth of the different inhabitants of this planet that there is scarcely one single common element or character by which we may surely and constantly aver that the body we have up for examination is certainly a tooth if it happen to be in the stage of evolution prior to calcification or solidification.

And even the various hard constituents of teeth are not freed from ambiguity by our best efforts with our best glasses. For the mergence of enamel into dentine and this into cement and cement into bone is so gradual as to defy our sharpest tests up to the present time, unless we be well acquainted with the particular class of animal to which they belong. So, if the certainty depends upon our acquaintance with analagous bodies you perceive that it is the analogy, and not the particular structure, which enables us to decide to what form of creature to refer it.

Then if all certainty depends upon the unknown, is it not clear that negation is quite equal to affirmation! or that 0 is equal to 1? or theory to practice or infinity to eternity or God to creation!!

Now that all this is infinitessimally contained in, suggested, and foreshadowed by the evolution of a single mammalian tooth I do not hope that all will see at first. But, nevertheless, it is positively so and we *must* grasp things synthetically first if we would grasp them at all. Even when we think we attain our knowledge by an item at a time, what we call item is the synthesis of one aspect of the whole subject under consideration.

So if we must take synthetically all cur textual statements, let us have our text as comprehensive as possible to fecilitate our labors.

Nearly the whole world have sought the final solution of the problems of creation, development and growth by the observations of the intellect without the aid of direct independent inspiration and hence the fractional doctrines that have held the dominion over the minds of the best and most earnest searchers after the complete solution of the problem of creation!

To all barely analytical thinkers this will necessarily remain so, until exalted perception shall open the spiritual *type* of rock, shell and tree; radiate, articulate and vertebrate in its first form, after creation, before it has found time and means to express itself in bodily material shape.

All because they persistently deny that inspiration is the prime prerequisite to perception at all whether by the channel of ordinary sense or an exalted clairvoyance.

The very terms, develop and evolve, hold the truth in an enfolded state, precisely as the type does the future tooth or other material body. Evolution and development unroll and bring within the perview of our senses the *plan* of the creation of things; among which the teeth are of prominent interest to dentists for obvious reasons.

All that I have said of bodies in general might be said of a single mammalian tooth, to understand which to the fullest extent implies completeness of knowledge, in all other products of the creative procedure.

The granular, *follicular* and *sacular* stages of development of teeth are but the degrees of accomplishment of the work of type and material as they progress toward a fitness of the tooth for the destined use in the animal economy.

In case the organizing force be withheld on any point in these labors the results will be imperfect, despite the most perfect plan of properly prepared materials out of which to construct these organs so necessary to the due comminution of the food for the growth and maintenance in vigor of the body to which they belong.

And on the other hand the most perfect typal force is powerless to produce perfect teeth or other organs without adequately prepared materials upon which to express its morphological power.

Therefore, it behooves us to look very closely into these necessary elemental conditions that we may be the better able to detect their harmonious or inharmonious workings, and thereby secure well-developed organs to those in our charge at this important period of their existence.

For if we shall have secured a perfect set of teeth to those in our care we have gained nine-tenths of the vantage ground for immunity from suffering, long and useful life.

If then all failures to accomplish these desirable results are the consequence of the prevailing ignorance among parents and guardians who have children under their care, and the more criminal ignorance of the dental profession rendering them unable to guide the anxious inquirers of "what shall we do to secure our children from the wholesale loss of their teeth at an early age rendering it necessary to have artificial substitutes or go without? Does it not call loudly on each and all who have been fortunate enough to see the way out of this slough of despond to set about a prompt reformation of the entire knowledge and practice of the profession and people, and interrogate madam nature so earnestly and persistently as to set us above our former sins and ignorances and render us what we ought to be, the real conservators of the lives and the health of the communities in which our lots are cast.

And how else shall we do this so effectually as to associate intimately and fraternally, each communicating of his best efforts until we shall all be corrected by the kindly contacts and criticisms fully up to complete competency to fulfill all the reasonable demands of an enlightened and very needy community.

